# Pilot Assessment of Brain Metabolism in Perinatally HIV-Infected Youths Using Accelerated 5D Echo Planar J-Resolved Spectroscopic Imaging

**DOI:** 10.1371/journal.pone.0162810

**Published:** 2016-09-13

**Authors:** Zohaib Iqbal, Neil E. Wilson, Margaret A. Keller, David E. Michalik, Joseph A. Church, Karin Nielsen-Saines, Jaime Deville, Raissa Souza, Mary-Lynn Brecht, M. Albert Thomas

**Affiliations:** 1 Department of Radiology, David Geffen School of Medicine, University of California Los Angeles, Los Angeles, CA, 90095, United States of America; 2 Los Angeles Biomedical Research Institute at Harbor-UCLA Medical Center, Department of Pediatrics, Torrance, CA, 90502, United States of America; 3 Department of Pediatrics, David Geffen School of Medicine, University of California Los Angeles, Los Angeles, CA, 90095, United States of America; 4 Department of Pediatrics, Miller Children’s Hospital at Long Beach, Long Beach, CA, 90806, United States of America; 5 Department of Pediatrics, Children’s Hospital Los Angeles, Los Angeles, CA, 90027, United States of America; 6 Department of Behavioral Sciences, David Geffen School of Medicine, University of California Los Angeles, Los Angeles, CA, 90095, United States of America; Linköping University, SWEDEN

## Abstract

**Purpose:**

To measure cerebral metabolite levels in perinatally HIV-infected youths and healthy controls using the accelerated five dimensional (5D) echo planar J-resolved spectroscopic imaging (EP-JRESI) sequence, which is capable of obtaining two dimensional (2D) J-resolved spectra from three spatial dimensions (3D).

**Materials and Methods:**

After acquisition and reconstruction of the 5D EP-JRESI data, T_1_-weighted MRIs were used to classify brain regions of interest for HIV patients and healthy controls: right frontal white (FW), medial frontal gray (FG), right basal ganglia (BG), right occipital white (OW), and medial occipital gray (OG). From these locations, respective J-resolved and TE-averaged spectra were extracted and fit using two different quantitation methods. The J-resolved spectra were fit using prior knowledge fitting (ProFit) while the TE-averaged spectra were fit using the advanced method for accurate robust and efficient spectral fitting (AMARES).

**Results:**

Quantitation of the 5D EP-JRESI data using the ProFit algorithm yielded significant metabolic differences in two spatial locations of the perinatally HIV-infected youths compared to controls: elevated NAA/(Cr+Ch) in the FW and elevated Asp/(Cr+Ch) in the BG. Using the TE-averaged data quantified by AMARES, an increase of Glu/(Cr+Ch) was shown in the FW region. A strong negative correlation (r < -0.6) was shown between tCh/(Cr+Ch) quantified using ProFit in the FW and CD4 counts. Also, strong positive correlations (r > 0.6) were shown between Asp/(Cr+Ch) and CD4 counts in the FG and BG.

**Conclusion:**

The complimentary results using ProFit fitting of J-resolved spectra and AMARES fitting of TE-averaged spectra, which are a subset of the 5D EP-JRESI acquisition, demonstrate an abnormal energy metabolism in the brains of perinatally HIV-infected youths. This may be a result of the HIV pathology and long-term combinational anti-retroviral therapy (cART). Further studies of larger perinatally HIV-infected cohorts are necessary to confirm these findings.

## Introduction

The human immunodeficiency virus (HIV) can affect the central nervous system (CNS) directly through infection, as well as indirectly by suppressing the immune system and allowing for opportunistic infection in both perinatally HIV-infected children and those who acquire HIV infection as adolescents or adults. In addition, the possible central nervous system effects of long term anti-retroviral therapy must be considered. Perinatally HIV-infected youths are a unique population because they acquired HIV infection at a time of immune compromise *in utero* or at birth, and signs of CNS involvement have been well documented [1, 2]. However, with advances in combinational antiretroviral therapy (cART), the incidence rate of HIV encephalopathy in these perinatally HIV-infected patients has greatly declined [3]. Nonetheless, monitoring the progression of HIV is important in order to ensure early detection of neurocognitive disorders or other CNS abnormalities arising from the HIV infection and its treatment.

One method capable of discerning metabolic changes *in vivo* is ^1^H magnetic resonance spectroscopy (MRS). Cerebral one dimensional (1D) MRS has previously been used to investigate the differences between healthy children and pediatric patients with HIV [4–9]. These studies were performed using the conventional point-resolved spectroscopy (PRESS) sequence [10], which is capable of localizing a volume of interest and measuring key metabolites including N-acetylaspartate (NAA), creatine (Cr), choline (Ch), and myo-Inositol (mI). A common finding between these studies was the decreased NAA/Cr ratio in perinatally HIV-infected patients with HIV encephalopathy. NAA is a marker used to measure neuronal integrity, and a decrease in concentration is indicative of neuronal loss. Additionally, two dimensional (2D) MRS has also been used to assess the differences between healthy and HIV-infected children [11, 12]. The localized correlated spectroscopy (L-COSY) sequence employed in these studies is a powerful technique capable of spreading overlapping resonances into a second dimension, therefore providing a means to reliably quantify more metabolites [13]. However, due to the introduction of the time increment (t_1_) which is necessary for detection of cross peaks between metabolite protons with J-coupling, the L-COSY sequence is very time consuming. This is true of other 2D techniques as well, including the J-resolved spectroscopy (JPRESS) sequence [14–16], which uses a time increment to detect J-modulation. Unfortunately, while these single voxel 1D and 2D techniques provide a wealth of information from a single location, they are unable to acquire metabolic information from multiple spatial locations in the brain.

Supplementing this deficiency, ^1^H magnetic resonance spectroscopic imaging (MRSI) [17] is capable of providing metabolic information from numerous spatial locations in a single scan at the expense of longer acquisition duration. The MRSI sequence can be accelerated by using an echo planar bipolar gradient train for readout along one spatial dimension, eliminating the need for phase encoding along that dimension [18, 19]. Combining this spatial acquisition with 2D JPRESS spectral acquisition, called echo planar J-resolved spectroscopic imaging (EP-JRESI) [20], allows for acquisition of J-resolved spectra from multiple spatial regions. However, the disadvantage of the EP-JRESI sequence results from the numerous phase encoding and time increments necessary for acquisition, which once again lead to longer scan times. This disadvantage can be overcome by applying non-uniform sampling to the incremented dimensions with compressed sensing reconstruction [21–24]. This method is applicable to four dimensional spectroscopic imaging as well as five dimensional spectroscopic imaging [25,26].

In particular, the five dimensional (5D) EP-JRESI sequence yields J-resolved spectra from three spatial dimensions. With further processing, this type of acquisition can also yield TE-averaged spectra [27, 28] from the same spatial dimensions, called TE-averaged echo planar spectroscopic imaging (TEA-EPSI). TE-averaged spectroscopy is a subset of J-resolved acquisition known for glutamate (Glu) detection, and has also been used to detect differences between healthy adults and adults with HIV [29, 30]. The purpose of this study was to evaluate changes between healthy youths and perinatally HIV-infected youths using the 5D EP-JRESI and TEA-EPSI techniques. The resulting J-resolved and TE-averaged spectra were quantified and the metabolite concentrations were compared to determine statistical differences between the two groups.

## Methods

### Subjects

Eight perinatally HIV-infected youths (mean age = 18.6 years) and seven healthy youths (mean age = 19 years) were investigated at the University of California–Los Angeles Medical Center. The imaging research protocol, written consenting procedure, and written consent forms were approved by the Institutional Review Boards at both UCLA and the Los Angeles Biomedical Research Institute at Harbor-UCLA Medical Center. If a subject was under the consenting age of 18, a parent or legal guardian signed the written consent forms on their behalf. The perinatally HIV-infected youths were referred from the HIV clinics at Harbor-UCLA Medical Center, Miller’s Children’s Hospital, Children’s Hospital of Los Angeles, and David Geffen School of Medicine at UCLA. The following inclusion criteria were used: subject was 13–25 years of age, subject acquired HIV from the mother *in utero* or at birth, subject was currently receiving cART, and subject was right-hand dominant. Exclusion criteria for HIV subjects were as follows: subject had any central nervous system diseases other than HIV including CNS opportunistic infections, subject had metallic implants, subject had claustrophobia, subject had attention deficit/hyperactivity disorder (ADHD), subject (female) was pregnant or not in the follicular phase of the menstrual cycle, subject used nicotine, alcohol, marijuana or other substances, and subject had any current psychiatric condition. In addition to the MR imaging studies performed, information on the HIV patients’ viral loads, CD4 cell counts, and medications were obtained from chart review. Information was also obtained regarding age of diagnosis and age at first treatment. Patient information is summarized in Table 1. One subject was referred as perinatally HIV-infected from Africa, but was discovered two years after the scan to not be perinatally HIV-infected.

**Table 1:** Relevant clinical information for all perinatally HIV-infected youths is shown.

Patient	Age at Scan (years)	Sex	CD4 count	Viral Loads	Treatment Duration (months)	HIV Encephalopathy
**1**	22	F	829	28	249	Yes
**2**	22	M	461	<20	102	Yes
**3**	19	M	1250	<20	218	Yes
**4**	18	F	355	212	165	No
**5**	17	F	319	2680	183	No
**6**	16	M	716	<20	191	No
**7**	18	F	626	<20	209	Yes
**8**	20	F	1011	<20	240	Probable

### Acquisition

All perinatally infected HIV and healthy subjects were scanned on a 3T scanner (Magnetom Trio-Trim, Siemens Medical Solution, Erlangen, Germany) using the 5D EP-JRESI protocol [25]. Localization of the PRESS volume of interest (VOI) [10] was based on a reference T_1_-weighted MRI scan. The 5D EP-JRESI parameters were as follows: TR/TE = 1200/30ms, detected (direct) spectral bandwidth = 1190 Hz, indirect spectral bandwidth = 500 Hz, (k_x_,k_y_,k_z_,t_2_,t_1_) = (16,16,8,256,64), FOV = 24x24x12cm^3^, and spatial resolution = 1.5x1.5x1.5cm^3^. A fully encoded acquisition would take 2.73 hours. However, the sequence was non-uniformly sampled to acquire only one-eighth of the total points (8x), resulting in an acquisition time of approximately 20 minutes for the metabolite scan, and 3 minutes for the water scan. For the water scan, only the first t_1_ point was acquired.

### Post-Processing

**Fig 1:**
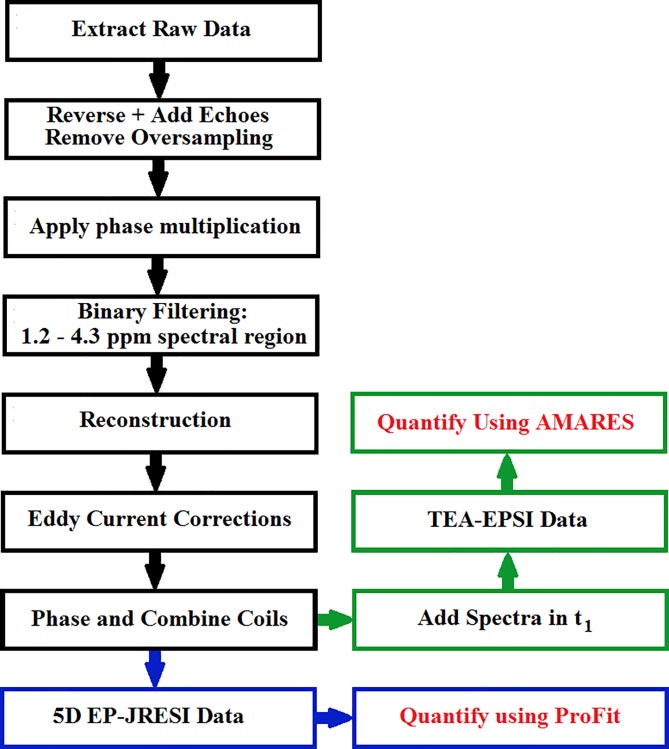
A diagram detailing the post-processing pipeline is displayed. The final results for the 5D EP-JRESI are highlighted in blue and the final results for the TEA-EPSI are highlighted in green. Red text is used to show the two quantitation methods.

The 5D EP-JRESI data and TEA-EPSI data were reconstructed as previously discussed [25, 28]. A diagram of the full post-processing and quantitation pipeline can be seen in Fig 1. First, the raw data were extracted and the odd echoes were time reversed. This was necessary to account for acquisition using the bipolar gradient for readout. Next, the odd and even echoes were summed, and spatial oversampling was removed in the readout direction. After performing a Fourier transformation on the direct spectral domain, a phase multiplication was applied to tilt the diagonal [31, 32]. Binary filtering was applied to the 1.2–4.3 ppm spectral in the (F_2_,F_1_) domain, which helped to reduce the dynamic range of the spectra [33]. Since binary filtering essentially replaces the lipid/water spectral regions with zeroes, the signal to noise ratio (SNR) and spectral resolution are unaffected within the 1.2–4.3 spectral range. After this process was performed, the data were reconstructed using the split Bregman [22, 34] algorithm, which was used to solve the following optimization problem on a coil-by-coil basis:

$$\underset{u}{\min}\;TV(u)\;s.t.\;{\left\|RFu-f\right\|}_{2}^{2}<\sigma^{2}$$ (1)

In Eq (1), *u*, *R*, *F*, f, and σ^2^ are the reconstructed data *u*(x,y,z,F_2_,F_1_), sampling mask, Fourier transform operator applied across (k_y_,k_z_,t_1_), undersampled data f(x,k_y_,k_z_,F_2_,t_1_), and estimate of the noise variance. *TV(u)* is the total variation of the reconstructed data. Total reconstruction time took approximately 45 minutes per coil on a 128 GB RAM workstation equipped with a 3.1 GHz Intel Xeon processor. After reconstruction, eddy current correction using the water scan was applied using Klose's correction [35]. The coils were then phased in the (F_2_,F_1_) domain by employing a non-linear least squares optimization to match the phases of the experimental data with the phases of the prior-knowledge NAA, Cr and Ch peaks [28, 36]. After the coils were phased individually, the coils were summed together, resulting in 5D EP-JRESI data. Even using eight-fold acceleration, 80% of the total SNR is retained using this method [25]. Finally, in order to obtain the TE-Averaged data, the 5D EP-JRESI data were inverse Fourier transformed to the (F_2_,t_1_) domain and the spectra along the t_1_ dimension were summed.

### Quantitation Regions

All HIV patients and healthy controls first had several brain regions identified based on T_1_-weighted MRI. Five representative voxels from the central slice were assigned as the right frontal white (FW), medial frontal gray (FG), right basal ganglia (BG), right occipital white (OW), and medial occipital gray (OG) regions for each subject. Since the voxel size was 1.5x1.5x1.5cm^3^, each representative voxel was a mixture of white and gray matter. However, the FW and OW were mostly white matter, and the FG and OG were predominantly gray matter. White matter voxels were chosen to the right of the medial gray matter voxels for all subjects, and the basal ganglia voxel was chosen to the right of the ventricles. These voxels were identical for both datasets acquired by the 5D EP-JRESI and TEA-EPSI techniques.

#### 5D EP-JRESI Quantitation

The updated ProFit [36] was implemented for quantifying the J-resolved spectra from the 5D EP-JRESI sequence. The following GAMMA simulated [37] metabolites were included as prior-knowledge for fitting: Cr3.0, Cr3.9, NAA, phosphocholine (PCh), Ch, aspartate (Asp), γ-aminobutyric acid (GABA), glucose (Glc), glutamine (Gln), glutamate (Glu), Glx (Glu+Gln), glutathione (GSH), lactate (Lac), myo-Inositol (mI), N-acetylaspartyl glutamate (NAAG), phosphoethanolamine (Pe), taurine (Tau), threonine (Thr), scyllo-Inositol (Scy), alanine (Ala), glycerylphosphorylcholine (Gpc), glycine (Gly), and ascorbic acid (Asc). ProFit uses an iterative approach to quantify metabolites by increasing the degrees of freedom with each outer iteration, including the number of metabolites fit, the line broadening of individual metabolites, and the frequency drift of individual metabolites. Estimation of these parameters using ProFit results in an accurate representation of the 2D J-resolved spectrum, and therefore an accurate concentration value for each metabolite [38]. The goodness of fit metric typically used in ProFit, the Cramer Rao Lower Bound % (CRLB%) [36], was not used stringently in this study as a quality control metric, since it is still unclear how non-uniform sampling and non-linear reconstruction affect this metric. Instead, the residuals from ProFit fitting were quantitatively assessed and used to exclude lower quality voxels. This quantitative approach was employed by first calculating the integrals of the NAA, Cr3.0, and PCh+Ch peaks from both the actual spectrum and the ProFit residual spectrum. The spectral regions used for integration were: NAA (1.95–2.05ppm, ±8Hz), Cr3.0 (2.95–3.05ppm, ±8Hz), and PCh+Ch (3.15–3.25ppm, ±8Hz). Then, a ratio was obtained between these two integral values with the residual integral value in the numerator and the actual integral value in the denominator for all three metabolites. Finally, if the average of these three ratios exceeded a threshold value of 0.15, the voxel was deemed as low quality and was excluded from statistical analysis. The threshold value was chosen after examining several high and low quality voxels. It is important to note that every voxel meeting this requirement was included in the study, even if metabolites in a particular voxel had high CRLB% values (>50%).

#### TEA-EPSI Quantitation

For TEA-EPSI quantitation, the advanced method for accurate robust and efficient spectral fitting (AMARES) [39] was used through the java-based magnetic resonance user interface (jMRUI) [40] package. The metabolites of interest for TEA-EPSI were mainly Glu and mI. A basis set was created for AMARES that incorporated the major brain metabolites (NAA, Glu, Cr, Ch, mI and Glx) and also considered the unique lineshapes that arise from the TE-averaging process. Lorentzian line shapes were chosen for all prior knowledge signals. For line broadening, each metabolite was allowed to vary from 1Hz to 5Hz. The prior knowledge accounted for any subtle frequency drifts by shifting ±0.01 ppm and ±0.02 ppm depending on the resonance. After quantitation, signal amplitudes corresponding to the same metabolite were summed, resulting in a total concentration value for each metabolite. Finally, this value was multiplied by a factor accounting for the proton number contributing to each of the resonances.

### Statistical Analysis

After the two methods were used for metabolite evaluation, statistical comparisons were made between the healthy and HIV youths. First, the means and standard deviations were calculated for each metabolite in each brain region of interest. Afterwards, a Student's two-tailed t-test was used to assess the differences between the two groups. Applying a modified Hochberg multiplicity 7 adjustment [41] for multiple t-tests performed, the adjusted significance level was defined as p < 0.013 in order to maintain a false discovery rate of .05 for tests across 2 measurement methods for 9 major metabolites within a brain region. Since Cr3.0 concentration may be altered in the HIV pathology, metabolite concentrations were also compared using Cr3.0 and total choline (Cr+Ch) as a reference. While this new denominator does not entirely mitigate the effects of Cr3.0 variation, it does ease the burden on Cr3.0 as the sole internal reference. Furthermore, to assess treatment effectiveness, the major metabolites measured with respect to (Cr+Ch) were correlated to both CD4 counts and duration of treatment for the HIV patients. A strong positive correlation was defined as greater than r = 0.6 and a strong negative correlation was defined as less than r = -0.6, where r is the correlation coefficient. Data analysis was performed including and excluding the patient that was not perinatally HIV-infected. Since both analyses demonstrated nearly identical findings, the patient was included in the study.

## Results

**Fig 2:**
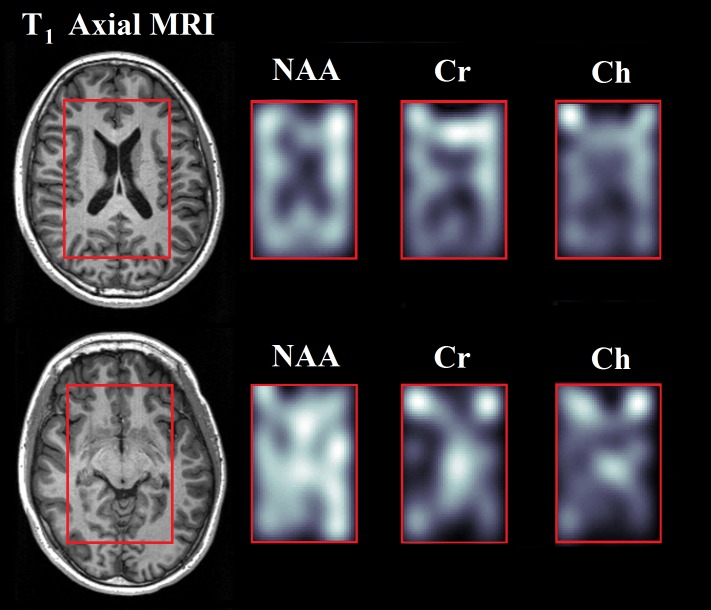
Metabolite maps of NAA, Cr, and Ch from a 16 year old HIV patient are shown. Two T_1_-weighted axial MRI from representative slices are shown on the left. The red box overlaid on the axial images represents the PRESS localization within the slice.

Metabolite images of NAA, Cr, and Ch alongside representative T_1_-weighted axial MRIs recorded in a 16 year old HIV patient can be seen in Fig 2. Two slices are displayed, and the PRESS localization in the axial plane is depicted by a red box. The metabolite images were reconstructed by using peak integration for the three singlets from the J-resolved spectra: total NAA (NAA + NAAG)–tNAA (1.9–2.1ppm, ±7.8Hz), Cr (2.9–3.1ppm, ±7.8Hz), and total Choline (PCh + Ch + Gpc)—tCh (3.1–3.3ppm, ±7.8Hz). For display purposes, all metabolite maps were zero filled in both the k_x_ and k_y_ dimensions when displaying each slice to increase spatial resolution and were truncated to the volume of interest (VOI). As expected, a lack of signal for all three metabolites is present in the ventricle area due to the large amount of cerebrospinal fluid (CSF) present. The second slice has hyperintense signal for Cr and Ch in the frontal left and frontal right regions, which may be a result of poor water suppression in these areas. Also, the Cr and Ch left-right asymmetry in this second slice may be caused by bulk motion during the scan combined with chemical shift displacement error (CSDE). Overall, the metabolite maps in conjunction with the T_1_-weighted MRI allowed for accurate labelling of the five voxels of interest, which were ultimately quantified.

The 5D EP-JRESI metabolite ratios quantified using ProFit with respect to Cr3.0, as well as (Cr+Ch) are tabulated in Tables 2 and 3. ProFit found significant differences (p < 0.013) in the FW and BG when compared to the healthy youths. HIV youths showed increased tNAA/Cr in the FW and increased Asp/Cr in the BG. Furthermore, trends (p < 0.05) were also present, including decreased Scy/Cr in the OG and increased Gln/(Cr+Ch) in the FW. Unfortunately, due to water contamination and the low spectral resolution, mI and other metabolites were not reliably quantifiable using ProFit. For mI specifically, the 4.1ppm resonance was very close to the filtering limit of 4.3ppm, and the presence of the residual water peak further hindered reliable quantitation.

**Table 2:** Quantitative results of the right frontal white (FW), medial frontal gray (FG), and right basal ganglia (BG) regions using ProFit are shown with respect to Cr3.0, as well as with respect to (Cr + Ch). Significant (p < 0.013) differences are denoted with an asterisk (*).

ProFit	** **	**FW**	** **	** **	**FG**	** **	** **	**BG**	** **
**Metabolite wrt Cr3.0**	**Healthy**	**HIV**	**p value**	**Healthy**	**HIV**	**p value**	**Healthy**	**HIV**	**p value**
**tNAA**	1.35 ± 0.19	1.68 ± 0.12	0.006*	1.39 ± 0.36	1.58 ± 0.22	0.268	1.44 ± 0.30	1.66 ± 0.35	0.266
**tCh**	0.38 ± 0.06	0.36 ± 0.07	0.639	0.35 ± 0.11	0.32 ± 0.09	0.582	0.36 ± 0.06	0.38 ± 0.11	0.726
**Glx**	1.30 ± 0.28	1.66 ± 0.67	0.423	2.01 ± 0.36	1.47 ± 0.54	0.159	0.84 ± 0.36	1.99 ± 0.98	0.063
**Glu**	0.91 ± 0.36	1.11 ± 0.56	0.602	1.49 ± 0.40	0.92 ± 0.43	0.089	0.45 ± 0.43	1.50 ± 0.91	0.074
**Gln**	0.41 ± 0.10	0.53 ± 0.07	0.063	0.46 ± 0.10	0.55 ± 0.14	0.210	0.41 ± 0.11	0.48 ± 0.09	0.193
**Asp**	0.71 ± 0.43	0.60 ± 0.25	0.639	0.44 ± 0.13	0.46 ± 0.17	0.910	0.37 ± 0.02	0.62 ± 0.11	0.002*
**GABA**	0.57 ± 0.01	0.40 ± 0.16	0.297	0.32 ± 0.11	0.29 ± 0.13	0.829	0.37 ± 0.35	——	——
**GSH**	0.36 ± 0.07	0.28 ± 0.23	0.531	0.33 ± 0.18	0.27 ± 0.16	0.548	0.16 ± 0.03	0.23 ± 0.11	0.366
**Scy**	——	0.09 ± 0.04	——	0.14 ± 0.06	0.08 ± 0.07	0.431	——	0.06 ± 0.02	——
ProFit	** **	**FW**	** **	** **	**FG**	** **	** **	**BG**	** **
**Metabolite wrt (Cr + Ch)**	**Healthy**	**HIV**	**p value**	**Healthy**	**HIV**	**p value**	**Healthy**	**HIV**	**p value**
**tNAA**	0.98 ± 0.16	1.23 ± 0.11	0.012*	1.03 ± 0.29	1.21 ± 0.21	0.228	1.06 ± 0.23	1.20 ± 0.26	0.331
**tCh**	0.28 ± 0.03	0.26 ± 0.04	0.621	0.25 ± 0.05	0.24 ± 0.05	0.581	0.26 ± 0.03	0.27 ± 0.06	0.788
**Glx**	0.96 ± 0.23	1.23 ± 0.48	0.405	1.43 ± 0.27	1.14 ± 0.48	0.365	0.62 ± 0.28	1.48 ± 0.72	0.061
**Glu**	0.67 ± 0.28	0.82 ± 0.40	0.597	1.07 ± 0.31	0.72 ± 0.38	0.206	0.33 ± 0.32	1.11 ± 0.67	0.073
**Gln**	0.30 ± 0.07	0.39 ± 0.04	0.038	0.34 ± 0.07	0.42 ± 0.12	0.157	0.30 ± 0.08	0.35 ± 0.08	0.261
**Asp**	0.53 ± 0.34	0.44 ± 0.19	0.623	0.34 ± 0.10	0.35 ± 0.12	0.905	0.27 ± 0.02	0.45 ± 0.09	0.004*
**GABA**	0.42 ± 0.03	0.30 ± 0.14	0.341	0.24 ± 0.09	0.23 ± 0.11	0.901	0.28 ± 0.27	——	——
**GSH**	0.27 ± 0.05	0.21 ± 0.16	0.527	0.24 ± 0.12	0.20 ± 0.11	0.595	0.12 ± 0.02	0.16 ± 0.03	0.321
**Scy**	——	0.06 ± 0.02	——	0.09 ± 0.03	0.06 ± 0.05	0.466	——	0.04 ± 0.02	——

**Table 3:** Quantitative results of the right occipital white (OW) and medial occipital gray (OG) regions using ProFit are reported with respect to Cr3.0, as well as with respect to (Cr + Ch). No significant (p < 0.013) differences were found in these regions.

ProFit	** **	**OW**	** **	** **	**OG**	** **
**Metabolite wrt Cr3.0**	**Healthy**	**HIV**	**p value**	**Healthy**	**HIV**	**p value**
**tNAA**	1.78 ± 0.29	1.69 ± 0.40	0.636	1.73 ± 0.20	1.87 ± 0.17	0.199
**tCh**	0.37 ± 0.12	0.39 ± 0.14	0.776	0.31 ± 0.07	0.27 ± 0.07	0.301
**Glx**	1.60 ± 0.40	1.46 ± 0.63	0.635	2.57 ± 0.87	1.94 ± 0.66	0.170
**Glu**	0.89 ± 0.69	0.93 ± 0.56	0.888	1.97 ± 1.06	1.54 ± 0.65	0.356
**Gln**	0.58 ± 0.28	0.53 ± 0.18	0.668	0.40 ± 0.23	0.40 ± 0.12	0.986
**Asp**	0.59 ± 0.27	0.51 ± 0.27	0.580	0.61 ± 0.30	0.59 ± 0.25	0.867
**GABA**	0.38 ± 0.12	0.57 ± 0.41	0.585	——	0.19 ± 0.12	——
**GSH**	0.26 ± 0.11	0.13 ± 0.07	0.054	0.13 ± 0.04	0.10 ± 0.04	0.283
**Scy**	0.05 ± 0.03	0.05 ± 0.02	0.871	0.09 ± 0.05	0.04 ± 0.02	0.015
ProFit	** **	**OW**	** **	** **	**OG**	** **
**Metabolite wrt (Cr + Ch)**	**Healthy**	**HIV**	**p value**	**Healthy**	**HIV**	**p value**
**tNAA**	1.29 ± 0.14	1.20 ± 0.20	0.339	1.35 ± 0.14	1.47 ± 0.10	0.099
**tCh**	0.26 ± 0.07	0.27 ± 0.08	0.832	0.23 ± 0.04	0.21 ± 0.05	0.300
**Glx**	1.19 ± 0.30	1.04 ± 0.46	0.496	1.96 ± 0.70	1.54 ± 0.53	0.234
**Glu**	0.66 ± 0.52	0.67 ± 0.41	0.943	1.51 ± 0.81	1.22 ± 0.53	0.433
**Gln**	0.43 ± 0.22	0.37 ± 0.11	0.529	0.30 ± 0.16	0.31 ± 0.09	0.881
**Asp**	0.43 ± 0.19	0.36 ± 0.21	0.521	0.46 ± 0.21	0.46 ± 0.19	0.999
**GABA**	0.31 ± 0.13	0.48 ± 0.41	0.631	——	0.15 ± 0.09	——
**GSH**	0.18 ± 0.08	0.10 ± 0.07	0.101	0.10 ± 0.03	0.08 ± 0.04	0.413
**Scy**	0.03 ± 0.02	0.04 ± 0.01	0.930	0.07 ± 0.03	0.03 ± 0.01	0.018

**Fig 3:**
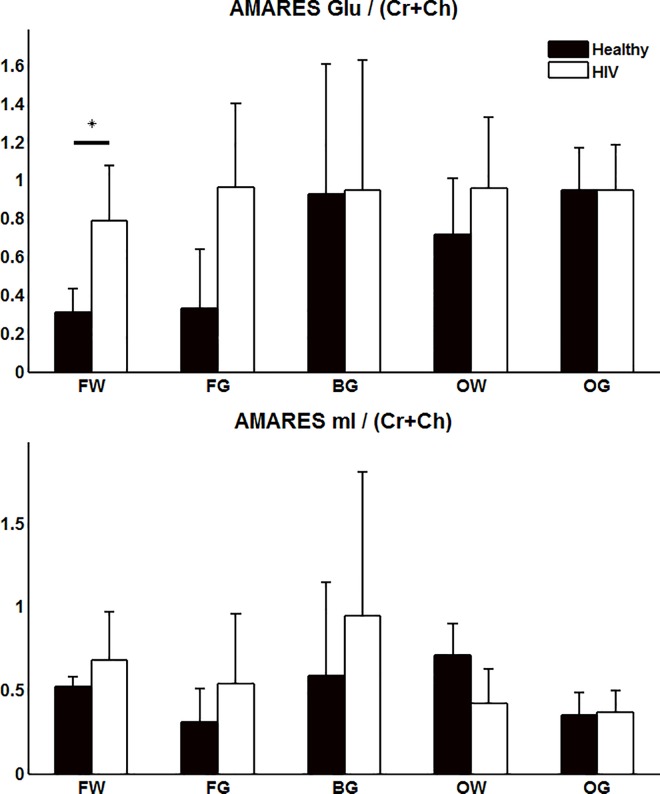
Metabolite ratios with respect to (Cr + Ch) are displayed for both Glu and mI using AMARES for quantitation. A significant (p < 0.013) difference, denoted with an asterisk, was found in the FW region for Glu.

Fig 3 shows the TE-averaged metabolite ratios quantified using AMARES of Glu and mI with respect to (Cr+Ch) as a reference. AMARES also found a significant difference between the two groups but only in one brain region. AMARES showed a significant increase of Glu in the FW when using (Cr+Ch) as a reference signal. Although not significant, the mI concentration of the HIV patients appears to be slightly elevated in the frontal region.

**Fig 4:**
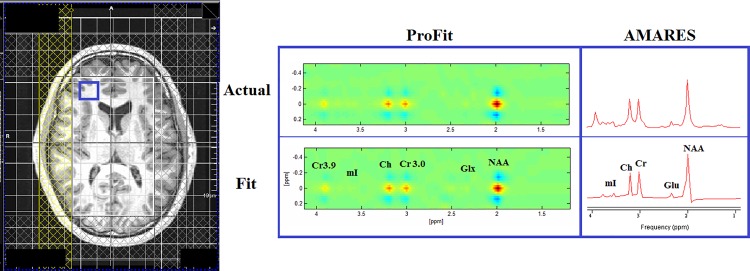
Axial MRI of a perinatally HIV-infected youth (age = 18 years) is shown on the left, where a frontal white matter voxel is highlighted in blue. The actual and fitted spectra from this voxel fit using both ProFit and AMARES are shown on the right.

**Fig 5:**
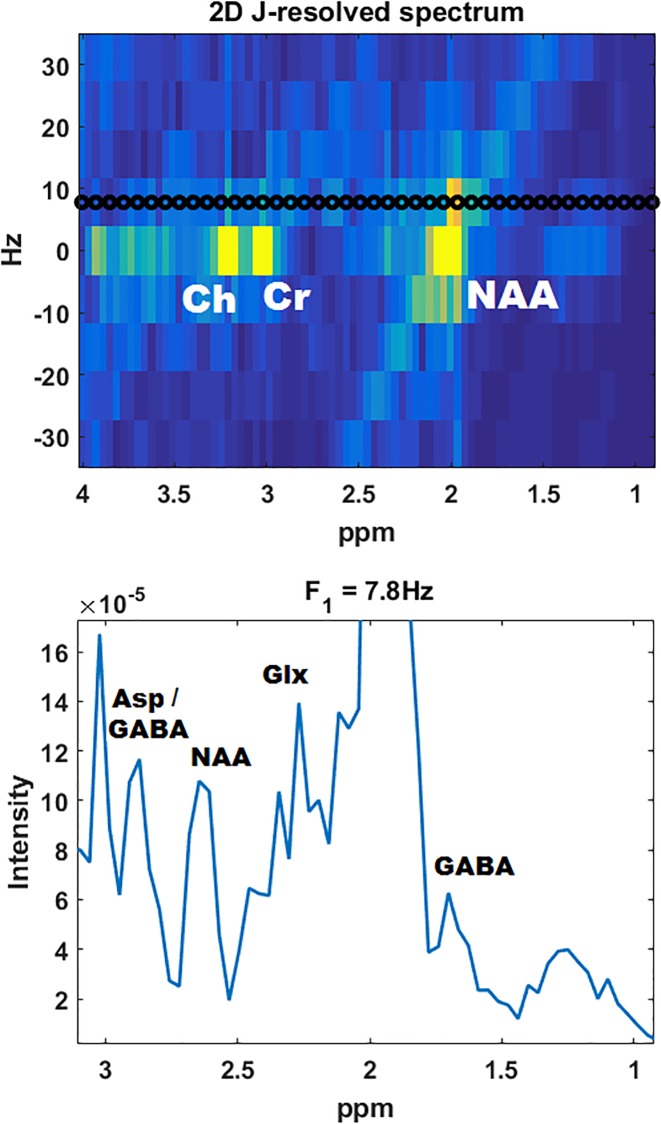
A 2D J-resolved spectrum is shown from a 22 year old HIV patient (top), and a one dimensional spectrum extracted at F_1_ = 7.8Hz line displayed using the black points is also shown (bottom). The crosspeaks of several lower concentration metabolites are visible at the expected spectral region.

A representative axial T_1_-weighted MRI along with J-resolved and TE-Averaged spectra from an 18 year old HIV patient are shown in Fig 4. Both ProFit and AMARES fit the actual spectra from the frontal white region well, as seen on the right of the figure. Inclusion of multiple peaks to represent a single metabolite proved useful for AMARES in order to accurately represent the unique lineshape resulting from the TE-averaged acquisition, where several different T_2_ weighted spectra are averaged together. By spreading the metabolite signals across a second spectral dimension, it is possible to view cross peaks of lower concentration metabolites, such as GABA, by using a one dimensional projection along the F_1_ dimension, as shown in Fig 5. ProFit automatically takes into account these cross peaks during quantitation and is able to identify metabolites even with the presence of F_1_ ridging.

Table 4 shows the sample correlation coefficients (r) between the major metabolites quantified using ProFit and the CD4 counts, as well as between the metabolites and the duration of treatment. Strong negative correlations are shown in the FW between tCh/(Cr+Ch) and CD4 count, as well as between tCh/(Cr+Ch) and treatment duration. In the FG region, strong positive correlations are shown between Asp/(Cr+Ch) and CD4 count, and also between Asp/(Cr+Ch) and treatment duration. Finally, the BG region showed a strong positive correlation between Asp/(Cr+Ch) and CD4 count. From AMARES, mI/(Cr+Ch) showed no correlation to either CD4 count or to treatment duration.

**Table 4:** Correlation values (r) between metabolite ratios quantified using ProFit and CD4 count, as well as between metabolite ratios and treatment duration are shown. The values greater than r = 0.6 or less than r = -0.6 are denoted with an asterisk (*).

Correlation Values (r)				
**ProFit Metabolite Ratio wrt (Cr+Ch)**	**FW**			
	**CD4 (r)**	**p value**	**Treatment (r)**	**p value**
**tNAA**	0.297	0.475	-0.028	0.947
**tCh**	-0.766*	0.027	-0.640*	0.088
**Glx**	0.425	0.294	-0.028	0.515
**Asp**	0.007	0.986	-0.159	0.707
** **	**FG**			
	**CD4 (r)**	**p value**	**Treatment (r)**	**p value**
**tNAA**	0.401	0.325	0.221	0.599
**tCh**	-0.034	0.935	-0.205	0.627
**Glx**	0.044	0.917	-0.081	0.849
**Asp**	0.752*	0.031	0.827*	0.011
** **	**BG**			
	**CD4 (r)**	**p value**	**Treatment (r)**	**p value**
**tNAA**	0.409	0.315	-0.473	0.236
**tCh**	-0.458	0.254	-0.342	0.407
**Glx**	0.301	0.469	-0.474	0.235
**Asp**	0.695*	0.055	-0.16	0.705
** **	**OW**			
	**CD4 (r)**	**p value**	**Treatment (r)**	**p value**
**tNAA**	-0.467	0.243	-0.581	0.131
**tCh**	-0.466	0.244	-0.21	0.618
**Glx**	-0.114	0.789	-0.588	0.126
**Asp**	-0.105	0.804	-0.545	0.162
** **	**OG**			
	**CD4 (r)**	**p value**	**Treatment (r)**	**p value**
**tNAA**	-0.233	0.578	0.103	0.808
**tCh**	0.119	0.779	0.314	0.449
**Glx**	-0.014	0.973	-0.225	0.592
**Asp**	0.347	0.4	0.274	0.512

## Discussion

Both the 2D J-resolved spectra and TE-Averaged 1D spectra obtained using the 5D EP-JRESI and TEA-EPSI techniques showed significant differences between healthy and perinatally HIV-infected youths. J-resolved spectra quantified using ProFit showed an increase in NAA/(Cr+Ch) in the FW and increase in Asp/(Cr+Ch) in the BG. TE-averaged spectra, a subset of the 5D EP-JRESI acquisition, quantified using AMARES showed increased Glu/(Cr+Ch) in the FW region. This additive finding may be attributed to the averaging process, where several TE lines are both constructively and destructively summed in order to highlight Glu. Due to the short TR (1200 ms) used in this study, significant T_1_ weighting implies that metabolite differences measured using the two methods are a product of both changes in T_1_ relaxation rates and differences in concentrations. Nonetheless, it is clear from these results that the perinatally HIV- infected youths receiving cART show a different energy metabolism in the brain than healthy youths.

**Fig 6:**
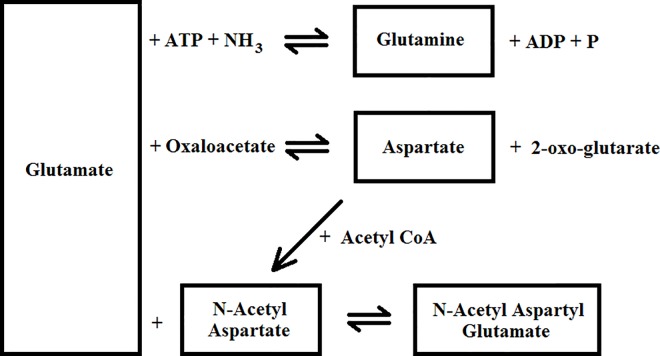
A simplified depiction of glutamate metabolism in the brain with respect to other metabolites (Gln, Asp, NAA, and NAAG) is shown [42–44].

Glutamate metabolism in the brain [42–44] is well studied and is greatly affected by HIV infection [45]. Fig 6 shows a simplified schematic diagram for the relationship between glutamate and other metabolites in the brain. Assuming the mechanisms for Glu transportation and uptake are unhindered, an increase in Glu can cause increases in Gln and Asp production, especially since Asp is favored in the equilibrium [46]. Asp also happens to be a direct precursor to NAA, and increased Asp may cause NAA to increase as well. The complimentary results from ProFit quantitation, where tNAA and Asp increase, and AMARES quantitation, where Glu is elevated, suggest that these metabolic changes stem from increased Glu. While neuronal integrity is intact, noted by the high tNAA concentration, it is not clear how these elevated metabolites may affect neuronal longevity and neurocognitive ability in the long term.

Previous MRS studies of perinatally HIV-infected patients have shown decreased NAA/Cr in different regions of the brain when neurological disorders were present [4, 6, 9], but no decrease in NAA/Cr was shown for perinatally HIV-infected children [7] in a population that included patients with and without signs of HIV encephalopathy. Of the 8 HIV patients in the current study: 4 had HIV encephalopathy, 1 had probable HIV encephalopathy, 2 had evidence of school difficulties (a recognized sign of HIV effects in the brain), and only 1 did not have evidence of CNS disease. Despite this high occurrence of CNS problems in our study population, no decrease in NAA was observed. Using L-COSY, an increase in the NAA/Cr cross-peak [11], an increase in the Glu/Cr diagonal peak [12], and also an increase in the Asp/Cr diagonal peak [12] have been shown for perinatally HIV-infected youths. These previous 2D spectroscopy findings support the ProFit and AMARES results of the current study. However, Glu/Cr has been shown to decrease in adult HIV patients with and without neurocognitive disorders [29–30, 47]. These different findings may be attributed to the T_1_ weighting of the spectra and the techniques employed to assess metabolic differences, as previously noted [12]. However, more strikingly, these findings suggest a possible difference in energy metabolism between perinatally HIV-infected youths and adults with HIV.

The differences in energy metabolism may also arise from the course of treatment and its effects on brain development. Seven of the eight pediatric patients examined in the current study have received antiretroviral therapy for the majority of their lives. This is in stark contrast to the HIV-infected adults that start treatment after reaching full maturity, when brain development is no longer in its crucial stages. MRS studies have examined the effects of cART on HIV-infected adults before and after treatment [48, 49], and a rising NAA/Cr ratio was noted in both studies. However, since it is not clear what the effects of prolonged cART are on perinatally HIV-infected youths, it is also unclear whether the increase of several metabolites (NAA, Glu, and Asp) in this study are a product of treatment, HIV pathology or a combination of the two.

In order to gain perspective on the relation of treatment effectiveness to metabolic changes, the major metabolites quantified using ProFit (tNAA, tCh, Glx, and Asp) were correlated to CD4 counts, which are representative of current immune system strength, and were also correlated to treatment duration. A negative correlation between tCh/(Cr+Ch) and CD4 count as well as treatment duration are in agreement with previous findings in adults [50], where higher Ch/Cr ratios were associated with lower CD4 counts. Increased Ch/Cr ratios have also been shown in AIDS dementia complex (ADC) patients in adults [51, 52]. Aspartate, on the other hand, showed a positive correlation with CD4 counts in both the FG and BG regions, and also showed a positive correlation to treatment duration in the FG. This may indicate that increased Asp is a result of treatment, and may shed light on the mechanism behind the observed increase of NAA after treatment in adults [48, 49]. Many 1D spectroscopy studies have not quantified Asp, and this is one metabolite of interest that 2D spectroscopy methods may be able to quantify more reliably.

One of the limitations of the current study is the small number of healthy youths and patients included in the two groups. Statistically significant metabolic differences were indeed found between the groups, however a larger cohort would allow for more elaborate statistical comparisons, and may help to elucidate the reasons behind the increase of several metabolites. Specifically, multivariate analysis may be able to determine a more comprehensive relationship between these metabolites throughout the five regions included in this study. The second limitation was the inability of ProFit to quantify mI reliably. The incorporation of better water suppression methods, such as VAPOR [53], would greatly improve mI detection by reducing the presence of a water tail. Better spectral resolution in the direct spectral domain may also allow for better detection of mI as well as other lower concentration metabolites. This can be accomplished by increasing the number of acquisition points from 256 to 512, with the caveat that this would drastically increase reconstruction time.

The third limitation of the study was the fact that ProFit results from each voxel needed to be assessed using a secondary method in order to evaluate acquisition and fit quality. This was necessary because the effects of compressed sensing reconstruction on CRLB% values are not well known, and thus CRLB% values were not used as a quality control metric. Voxel exclusion was only based on the voxel fit quality of NAA, Cr3.0 and PCh+Ch, so therefore voxels with high CRLB% values were still included in the study. By including results with all CRLB% values, quality filtering bias was not introduced into the results as recently described [54,55], and perhaps allowed for better discrimination between the healthy and patient groups. Finally, long scan durations and the presence of CSDE, as seen in Fig 2, are also limitations of the current technique and affect voxels within volume localization. Long scan durations allow for bulk motion and frequency drifts, and may cause voxel averaging over different anatomical regions of interest, further hindering gray and white matter separation. Adiabatic RF pulses [56] have been shown to greatly mitigate CSDE and can be incorporated into the 5D EP-JRESI sequence to improve overall performance and spatial localization. Even with all of these deficiencies, this study still shows results that are consistent with previous findings [11, 12] and demonstrates the potential uses of the 5D EP-JRESI technique to assess brain metabolic changes in HIV.

## Conclusion

The 5D EP-JRESI sequence was implemented in perinatally HIV-infected and healthy youths. The quantitation of J-resolved spectra using ProFit showed elevated tNAA/(Cr+Ch) in the frontal white region and elevated Asp/(Cr+Ch) in the basal ganglia region. Quantitation of TE-Averaged spectra using AMARES showed elevated Glu/(Cr+Ch) in the frontal white region. These complimentary results are indicative of an abnormal energy metabolism in the brain of the perinatally HIV-infected youths, and further studies are necessary to confirm these findings in a larger cohort of patients.
